# A FASTER Approach to Developing and Evaluating a Smart Insole Technology for Persons Living with Early‐Stage Dementia to Sustain Out‐Of‐Home Participation

**DOI:** 10.1002/alz70858_102458

**Published:** 2025-12-25

**Authors:** Nikki Meng Qi Zhang, Parnian Safaraifard, Amy Hwang, Thomas TANNOU, Rosalie H. Wang

**Affiliations:** ^1^ University of Toronto, Toronto, ON, Canada; ^2^ Centre de recherche de l'Institut universitaire de gériatrie de Montréal, Montreal, QC, Canada; ^3^ CRIUGM, CIUSS Centre‐Sud de l'Ile de Montréal, Montreal, QC, Canada

## Abstract

**Background:**

Persons living with dementia or cognitive impairment (PLWD/CI) often experience barriers to out‐of‐home participation (i.e., places outside home or in public) due to cognitive inaccessibility, risks or anxieties related to wandering, and care partners’ safety concerns – which can impede health and quality of life. Technology offers the potential to support PLWD/CI in sustaining out‐of‐home participation, but real‐world evaluations within complex, dynamic care ecosystems are necessary to ensure safety and effectiveness. The “Framework for Accelerated and Systematic Technology‐based intervention development and Evaluation Research” (FASTER) was created to support the development and evaluation of interventions from conception to real‐world use. The aim is to illustrate how FASTER was applied to a novel intervention encompassing a smart shoe insole technology designed to support out‐of‐home participation for PLWD/CI by enabling tracking, safety alerts, and care collaboration with care partners.

**Method:**

The FASTER approach includes the following phases: (1) Development, (2) Progressive Usability and Feasibility Evaluation, and (3) Phased Evaluation and Implementation. FASTER was applied to developing and evaluating the smart insole technology for PLWD/CI in real‐world use contexts.

**Result:**

Phase 1 collaborated with the commercial partner to conceptualize the intervention design vis à vis technology design features and use cases, and model theoretically‐ and evidence‐driven processes and outcomes. Six researchers conducted mixed‐method pilot usability testing by completing structured use cases in real‐world settings. Technical and design issues were identified and evaluation methods were refined to optimize usability, reliability, and mitigate safety risks, for subsequent evaluation with PLWD/CI and care partners. Phase 2 employed a small‐scale, mixed‐method multiple case study design to evaluate technology usability and feasibility, and primary outcomes (i.e., PLWD/CI's out‐of‐home participation, dyadic care collaboration) with 3 care ecosystems. Future research will adapt and scale the evaluation methodology for Phase 3, involving implementation and evaluation in a larger cross‐Canadian study in Quebec, Ontario, and British Columbia.

**Conclusion:**

FASTER is a systematic, iterative, and participatory approach to developing, evaluating, refining, and integrating technology‐based interventions. This presentation highlights the application of FASTER using a real‐world example, showcasing its value for both technological and health/social outcomes, including out‐of‐home participation and collaborative caring for PLWD/CI.

